# Nature-Inspired Green Procedure for Improving Performance of Protein-Based Nanocomposites via Introduction of Nanofibrillated Cellulose-Stablized Graphene/Carbon Nanotubes Hybrid

**DOI:** 10.3390/polym10030270

**Published:** 2018-03-06

**Authors:** Shicun Jin, Kuang Li, Jianzhang Li

**Affiliations:** 1 Key Laboratory of Wood Materials Science and Utilization, Beijing Forestry University, Ministry of Education, Beijing 100083, China; jinsc1994@bjfu.edu.cn (S.J.); kuangli@bjfu.edu.cn (K.L.); 2 Beijing Key Laboratory of Wood Science and Engineering, Beijing Forestry University, Beijing 100083, China; 3 College of Materials Science and Technology, Beijing Forestry University, Beijing 100083, China

**Keywords:** nanofibrillated cellulose, composite film, nanophase reinforcing, synergistic interactions

## Abstract

Soy protein isolate (SPI) provides a potential alternative biopolymer source to fossil fuels, but improving the mechanical properties and water resistance of SPI composites remains a huge challenge. Inspired by the synergistic effect of natural nacre, we developed a novel approach to fabricate high-performance SPI nanocomposite films based on 2D graphene (G) nanosheets and 1D carbon nanotubes (CNTs) and nanofibrillated cellulose (NFC) using a casting method. The introduction of web-like NFC promoted the uniform dispersion of graphene/CNTs in the biopolymer matrix, as well as a high extent of cross-linkage combination between the fillers and SPI matrix. The laminated and cross-linked structures of the different nanocomposite films were observed by field-emission scanning electron microscope (FE-SEM) images. Due to the synergistic interactions of π–π stacking and hydrogen bonding between the nanofillers and SPI chains, the tensile strength of SPI/G/CNT/NFC film significantly increased by 78.9% and the water vapor permeability decreased by 31.76% in comparison to neat SPI film. In addition, the ultraviolet-visible (UV-vis) light barrier performance, thermal stability, and hydrophobicity of the films were significantly improved as well. This bioinspired synergistic reinforcing strategy opens a new path for constructing high-performance nanocomposites.

## 1. Introduction

Sustainable and eco-friendly biopolymers, usually prepared from renewable resources such as proteins, polysaccharides, lignin and cellulose, have received a great deal of research attention as potential alternatives to conventional petrochemical-based materials [1,2]. Soy protein isolate (SPI), obtained as a by-product in the soy oil industry, is the most abundant plant-derived protein among all the natural biopolymers [3]. Due to its superior qualities of low cost, biodegradability, non-toxicity and easy availability, SPI-based film has been considered one of the most promising and ideal candidates for commercial applications such as food packaging [4], coating materials [5], tissue engineering [6], drug delivery [7] and air filtration [8]. However, pristine SPI-based films have some drawbacks such as poor mechanical properties and high sensitivity to moisture, which limit their further practical application [9]. Previous studies reported that a variety of methods could be used to optimize the functional properties of SPI-based materials, including physical treatment [10], chemical cross–linking [11], enzyme treatment [12], and block copolymerization [13].

Nanophase reinforcement is considered one of the most effective ways to improve the performance of biopolymer materials [14]. Nanostructured modification has emerged as a key research field in recent years due to its effectiveness in improving the properties of materials [15]. Nacre consists of 95 vol % two-dimensional (2D) inorganic platelets (calcium carbonate) and 5 vol % one-dimensional (1D) elastic biopolymers (chitin and protein). It has been successfully used to design many bio-based nanocomposite materials due to its high strength and toughness [16]. Graphene, a 2D layer of sp^2^ hybridized carbon atoms arranged in a honeycomb hexagonal lattice structure, is an ideal material to be used as a building block for designing and fabricating artificial nacre nanocomposites because of its extremely high specific surface area, superior thermal conductivity, and unique physicochemical performance [17]. There have been many studies on graphene-sheet modified polymer composites, including epoxy [18], poly(methyl methacrylate) (PMMA) [19], and polyethyleneimine (PEI) [20].

Carbon nanotubes (CNTs) are one of the 1D high-performance nanofillers. These have been extensively employed as a reinforcing and functional filler to improve the mechanical properties of polymer composites [21]. Gong et al. reported that the assembly of graphene with CNTs yielded hybrid materials with unique structural characteristics and diverse properties [22]. Shen et al. demonstrated that a graphene oxide/multi-walled carbon nanotube (MWCNT) hybrid could effectively improve the mechanical performance of epoxy composites [23]. However, carbon-based material is unstable in most solvents because of its chemical inertness and van der Waals interactions [24]. The main challenges of the traditional approaches were the poor dispersion of graphene in the polymer matrix, and the weak interface interactions between the filler and polymer [25]. As a strong and flexible 1D nanomaterial, nanofibrillated cellulose (NFC) possesses both high strength and stiffness, as well as having the qualities of being lightweight, transparency, biodegradability, biocompatibility, and remarkable reinforcing capabilities [26]. Song et al. found that NFC homogeneously embedded in the fillers and naturally adjusted their basal plane parallel to the surface, thus forming a continuous network [27]. Li et al. found that the introduction of NFC effectively prevented graphene oxide (GO) and CNTs from restacking and aggregating [28]. Therefore, NFC has been widely applied in the fabrication of high-performance materials with various fillers, such as montmorillonite [29], chitosan [30], and boron nitride nanosheets [31]. In our previous report, we demonstrated that the integration of PEI-modified cellulose nanocrystals (CNC) and graphene sheets into the SPI matrices resulted in a significant increase of mechanical properties via strong ion pairing between positively charged CNC and anionic graphene sheets [21]. However, this approach involved toxic chemical and polluted the water environment. In addition, the enhancement mechanism of the composite film was not explained clearly. To move toward a sustainable product requires a rethink to determine if a strategy that does not rely on toxic organic reagents could similarly improve the properties of biopolymer-based composites.

Inspired by the synergistic effect in the hierarchical structure of natural nacre, we have developed a novel approach to fabricating high-performance SPI nanocomposite films based on 2D graphene nanosheet and 1D CNTs and NFC using a casting method. The approach was feasible and green for building-enhanced interfacial interactions between the nanophase and SPI matrix. The web-like NFC was introduced to promote the uniform dispersion of carbon-based materials in the biopolymer matrix, as well as the high extent of cross-linkage combination between the fillers and SPI matrix. Furthermore, to understand the synergistic reinforcing effect from our nacre-like nanohybrids, a crack extension model was proposed. In particular, we reported a simple and green method for preparing stable aqueous graphene dispersion, which included the exfoliation and fragmentation of graphite into graphene in bovine serum albumin (BSA) solution using a sonication procedure. The production of graphene could be homogeneously dispersed in the aqueous solution and easily used in various practical applications. The structural characteristics, surface morphology, UV-visible light barrier ability, mechanical properties, water resistance and thermal stability of the resultant nanocomposites were analyzed and investigated in this study.

## 2. Materials and Methods

### 2.1. Materials

SPI powder (95% protein) was supplied by Yuwang Ecological Food Industry Co., Ltd. (Shandong, China). Graphite powder was obtained from Sinopharm Chemical Reagent Co., Ltd. (Shanghai, China). CNTs with a purity of 95 wt % were provided by Tianjin Heowns Biochem Co., Ltd. (Tianjin, China). NFC solution (solid content ~1%) was purchased from Tianjin Woodelf Biotechnology Co., Ltd. (Tianjin, China). BSA (99% purity) was supplied by Beijing Labest Bio Technology Co., Ltd. (Beijing, China). Glycerol (99% pure) and other chemical reagents of analytical grade were acquired from Beijing Chemical Reagents Company (Beijing, China).

### 2.2. Preparation of Stable Aqueous Graphene Dispersion

BSA powder (100 mg) was dispersed in 400 mL deionized water and constantly stirred at 50 °C for 10 h. The pH value of the BSA solution was adjusted to 3.0 with HCl solution. Then, 2 g of graphite powder was added into the pre-prepared BSA solution (400 mL) with the magnetical stir for 30 min at room temperature. Thereafter, the mixture was treated by sonication for 24 h and centrifuged at 3000 rpm for 30 min to obtain the supernatant of graphene dispersion.

### 2.3. Fabrication of Soy Protein Isolate (SPI) Based Nanocomposite Films

**Scheme 1:**
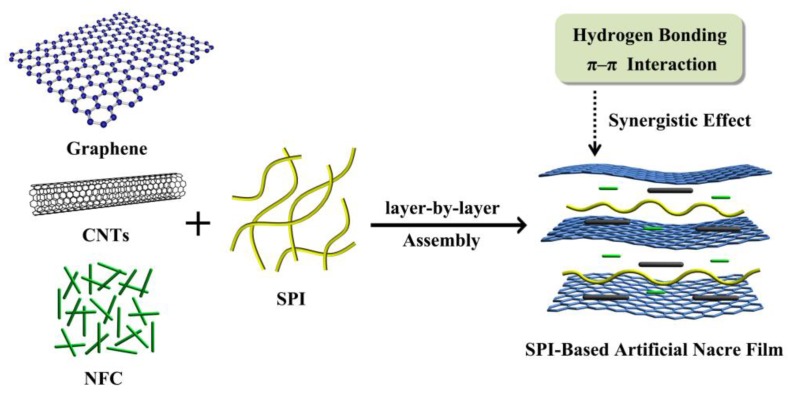
The schematic diagram of the fabrication procedure for SPI-based artificial nacre film.

For the control film, SPI powder (5 g) and glycerol (2.5 g) were added into deionized water (95 g) with a magnetic stirrer at room temperature. The pH of the mixture was adjusted to 9.0 with NaOH solution. Then, the obtained mixture was constantly stirred at 85 °C for 30 min. To fabricate SPI/CNT and SPI/NFC nanocomposite films, CNTs (0.01 g) and NFC (1 g, 1 wt %), respectively, were dispersed in the above solution (Table 1). The mixture was ultrasound-treated for 90 min and stirred constantly for 30 min. For the SPI/G nanocomposite film, the preparation process was the same as the control group, except that the deionized water was replaced with the prepared graphene dispersion. Based on SPI/G solution, CNTs (0.01 g) and NFC (1 g, 1 wt %) were sequentially dispersed in the above solution to obtain SPI/G/CNT/NFC mixture. Then, the mixture was ultrasound-treated for 90 min and stirred constantly for 30 min. Finally, the above solution was poured into a Teflon-coated plate and placed in a vacuum-dried oven at 45 °C for 24 h. All the films were stored in a controlled chamber (25 °C, 50% relative humidity) for 48 h before testing. The preparation mechanism of SPI-based nanocomposite film is illustrated in Scheme 1.

### 2.4. Characterization

The surface morphologies of graphene sheets and NFC were characterized by using an atomic force microscopy (AFM) analyzer (Bruker Multimode 8, Billerica, MA, USA). NanoScope Analysis software was used to analyze the AFM height images.

The dispersion morphology of CNTs was investigated by a transmission electron microscopy (TEM) analyzer (JEM-2100F, JEOL, Tokyo, Japan) at an acceleration voltage of 200 kV.

Attenuated total reflectance–Fourier transform infrared (ATR–FTIR) spectra and X-ray diffraction (XRD) patterns were used to analyze the chemical structure of SPI-based nanocomposite films. ATR–FTIR spectra were obtained with a Nicolet 6700 spectrometer (Thermo Scientific, Pittsburgh, PA, USA) ranging from 650 to 4000 cm^−1^ with a total of 32 scans. XRD patterns were obtained with a D8 advance diffractometer (Bruker AXS, Karlsruhe, Germany) scanned from 5° to 60° at 40 kV with a current of 40 mA.

The fracture surface morphologies of the nanocomposite films were observed by a scanning electron microscopy (SEM) analyzer (SU8010, Hitachi, Tokyo, Japan) at an accelerating voltage of 5 kV.

An ultraviolet-visible (UV-vis) spectrophotometer (TU-1901, Beijing Purkinje General, Beijing, China) was used to analyze the optical properties of SPI-based nanocomposite films ranging from 300 to 800 nm.

The tensile strength (TS), Young’s modulus (E), and elongation at break (EB) of SPI-based nanocomposite films were measured by using a tensile testing machine (INSTRON 3365, Norwood, MA, USA) at a loading speed of 50 mm/min. Five replicates (10 × 80 mm^2^) for each formulation were tested to obtain the standard deviation.

The thermal degradation behavior of SPI-based nanocomposite film was measured by a thermo-gravimetric (TG) analyzer (Q50, TA instruments, New Castle, DE, USA) under a 100 mL/min pure nitrogen flow rate from room temperature to 600 °C at a liner heating rate of 10 °C/min.

The water contact angles (WCA) were tested by using an OCA-20 contact angle apparatus (DataPhysics Instruments GmbH, Filderstadt, Germany) to investigate the surface hydrophobicity of SPI-based films. A sessile droplet (3 μL) of deionized water was dropped on the surface of the film and the WCA values were recorded at an interval of 0.1 s for 120 s. Five replicates were tested for each formulation.

The film sample (20 × 20 mm^2^) was placed in a desiccator (25 °C, 50% relative humidity) regulated with saturated K_2_CO_3_ solution for 48 h and weighed as (*m_a_*). The sample was then desiccated in a drying oven at 100 °C for 24 h and weighed as (*m_b_*). The moisture content (MC) values of the SPI-based films were calculated as follows:

MC (%) = (*m_a_* − *m_b_*)/*m_a_* × 100 (1)

Next, the sample was immersed in deionized water (100 mL) at room temperature for 24 h. The specimen was then oven-dried at 100 °C for 24 h and weighed as (*m_c_*). The total soluble matter (TSM) values of SPI-based films were calculated as follows:

TSM (%) = (*m_b_* − *m_c_*)/*m_b_* × 100 (2)

Finally, the sample was placed in P_2_O_5_-regulated desiccators (0% relative humidity) for 48 h and weighed as (*m_d_*). Then, the specimen was immersed in deionized water (100 mL) at room temperature for 24 h and weighed as (*m_e_*). The water absorption (WA) values of the SPI-based films were calculated as follows:

WA (%) = (*m_e_* − *m_d_*)/*m_d_* × 100 (3)

A water vapor permeability (WVP) tester (TSY–T1, Labthink Instruments, Jinan, China) was used to investigate the water vapor barrier abilities of SPI-based films (in accordance with ASTM E96). The samples were placed into a K_2_CO_3_-regulated desiccator (25 °C, 50% relative humidity) before testing. The WVP values of SPI-based films were calculated as follows:

WVP = WVTR·*x*/∆*p* (4)

where WVP represented the water vapor permeability (10^−1^·g·m^−1^·h^−1^·Pa^−1^); WVTR was the water vapor transmission rate (g·m^−2^·24·h^−1^); *x* was the thickness (m) of the film; ∆*p* was the water vapor pressure difference across the film (Pa).

## 3. Results and Discussion

### 3.1. Characterization of Graphene, Carbon Nanotubes (CNTs) and Nanofibrillated Cellulose (NFC)

**Figure 1:**
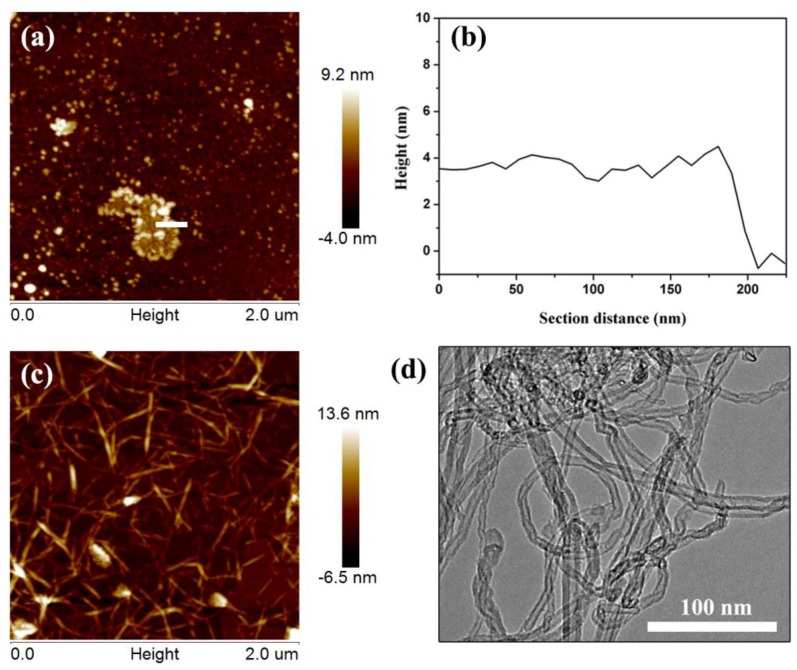
(**a**) Atomic force microscope (AFM) image, and (**b**) height profile image of the graphene nanosheet; (**c**) AFM image of NFC; (**d**) transmission electron microscope (TEM) image of CNTs.

The surface morphology characteristics of graphene, CNTs and NFC were investigated by AFM and TEM. The graphene nanosheet in the aqueous dispersion (Figure 1a) was clearly visible. The average diameter of the graphene nanosheet was approximately 500–1000 nm. The cross-sectional profile image was carried out to investigate the thickness of the prepared graphene, as shown in Figure 1b. The thickness of the graphene layer was about 4 nm, indicating the presence of multi-layer graphene sheets in the solution. The homogenous isolated nanofibers of NFC were observed in Figure 1c. The length and diameter of the NFC segments were approximately 500 nm and 10–20 nm, respectively. The TEM image of the CNTs was displayed in Figure 1d. The aggregated and bundled CNTs possessed hollow inner structures and their average diameter was approximately 10 nm.

### 3.2. Structural Analysis

**Figure 2:**
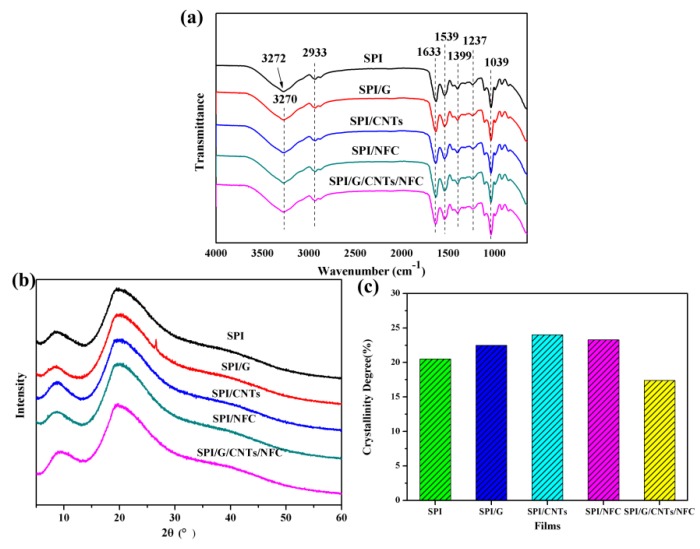
(**a**) Attenuated total reflectance–Fourier transform infrared (ATR–FTIR) spectra, (**b**) X-ray diffraction patterns, and (**c**) crystallinity parameters of SPI film and SPI-based nanocomposites.

The structural characteristics of the SPI-based nanocomposite films were analyzed by the ATR–FTIR spectra and XRD patterns. As shown in Figure 2a, the typical absorption peaks of SPI-based films at 1633 cm^−1^, 1539 cm^−1^, and 1237 cm^−1^ corresponded to amide I (C=O stretching), amide II (N–H bending), and amide III (C–N and N–H stretching), respectively [4]. The absorption peak of –CH_2_ group stretching vibrations was observed at 2933 cm^−1^ [32]. The absorption bands at 1399 cm^−1^ and 1039 cm^−1^ were attributed to the stretching vibrations of C–H and C–O, respectively [33]. In particular, the broad band around 3272 cm^−1^ corresponded to the O–H and N–H stretching vibrations, which were able to form hydrogen bonds with the carbonyl groups in the peptide linkage [3]. After the addition of graphene, CNTs, and NFC, the –OH stretching vibration of nanocomposite film shifted to a lower wave number at 3270 cm^−1^, which could be attributed to the increased hydrogen bonds in the composite that altered the absorbance of –OH vibration modes [6]. Therefore, hydrogen bonding might be the main interaction between the fillers and protein molecule chains, which promoted more interfacial interactions in the SPI matrix and improved the mechanical properties of nanocomposite films.

The XRD patterns were used to analyze the crystalline nature of SPI-based films. As seen in Figure 2b, the SPI-based films exhibited two major characteristic peaks at 2θ = 8.8° and 20.3°, which were attributed to the α-helix and β-sheet structures of the protein secondary structure, respectively [21]. The distinctive peak of the SPI/G film at 2θ = 26° corresponded to the (002) diffractions of graphitic carbon, which indicated the presence of graphene in the SPI matrix [34]. The crystalline structures of different nanocomposite films were investigated by measuring the crystallinity degree (Figure 2c). It could be observed that the SPI/G film had a higher crystallinity degree than the pure SPI film, which revealed that the structural order of protein conformation was obviously modified by graphene. In addition, the degree of crystallinity increased after incorporating CNTs, which was due to the peptide chains partially penetrated into the highly crystalline CNTs by orderly alignment [25]. Nevertheless, the SPI/G/CNT/NFC nanocomposite film showed a relatively low crystallinity degree, which was probably because the NFC promoted the uniform dispersion of the graphene and CNTs, as well as the high extent of cross-linkage combination between the fillers and SPI matrix [1].

### 3.3. Surface Morphology

**Figure 3:**
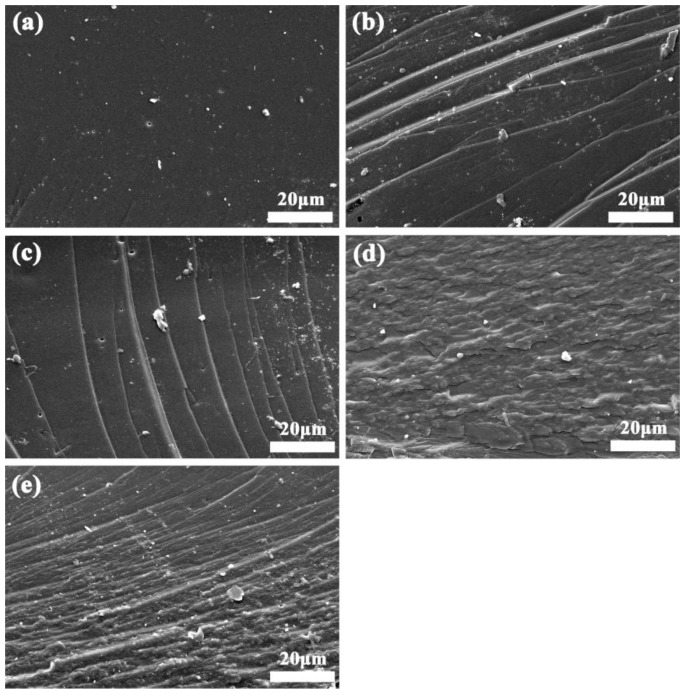
Scanning electron microscope (SEM) images of (**a**) SPI, (**b**) SPI/G, (**c**) SPI/CNT, (**d**) SPI/NFC, and (**e**) SPI/G/CNT/NFC nanocomposite films.

SEM images were obtained in order to study the cross-sectional surface morphology of the nanocomposite film. As shown in Figure 3a, the neat SPI film showed a relatively even and uniform fracture surface, suggesting the favorable biocompatibility and film-forming ability of the SPI molecules. The laminated structure of the SPI/G film (Figure 3b) indicated the enhanced interfacial adhesion between graphene nanosheets and SPI chains [35]. Compared with the SPI/G film, the SPI/CNT film showed a more rough surface with some aggregations (Figure 3c), which might be attributed to the agglomeration of CNTs that were assembled into the SPI matrix at a high concentration. After integrating NFC, the fracture surface of the SPI/NFC film became wrinkled and fluctuant (Figure 3d). This observation could be explained by the fact that the 1D nanofibrous NFC could act as extensional tentacles for the hybrid structures and was entangled with the polymer chains, which resulted in the enhancement of the interfacial interactions between the graphene/CNT and SPI matrix [28]. In particular, the SPI/G/CNT/NFC film exhibited a denser hierarchical construction (Figure 3e), which was due to the synergetic effect from graphene, CNTs, and NFC. This architecture with highly interlocked wrinkles could provide more available interfacial areas for the intimate interactions and promote stress transfer between fillers and polymer matrix, thus effectively improving the toughness and strength of the SPI-based nanocomposite films [36].

### 3.4. Dispersion of Graphene, CNTs and NFC

**Figure 4:**
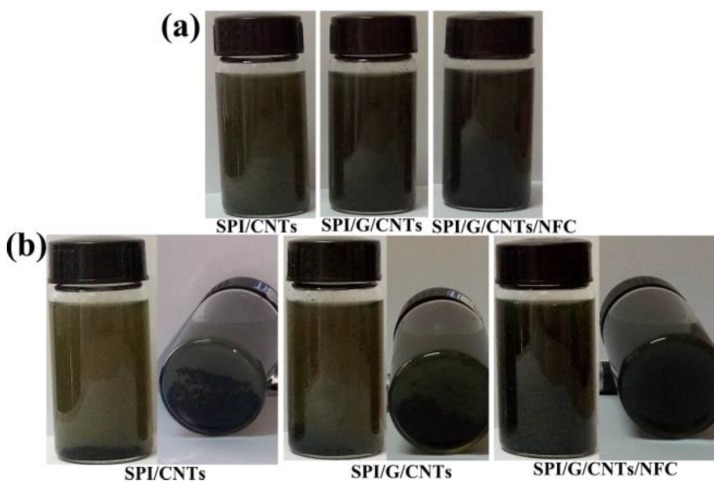
The dispersion of SPI/CNT, SPI/G/CNT, and SPI/G/CNT/NFC solutions after standing for (**a**) 1 min and (**b**) 2 h.

The dispersibility of graphene, CNTs and NFC in the SPI matrix is shown in Figure 4. The initial prepared solutions of different samples were dark and opaque (Figure 4a). After 2 h, it was observed that the CNTs were easily sedimented at the bottom of the SPI/CNTs and SPI/G/CNT bottles (Figure 4b) as a result of gravity [15]. Meanwhile, it is also worth noting that the NFC-assisted SPI/G/CNT dispersion exhibited good stability without obvious deposits in the bottom of this bottle. This could be explained by the hierarchical SPI/G/CNT/NFC architecture that inhibited the stacking of CNTs [37]. The above results demonstrated that the synergetic effect from graphene, CNTs and NFC on dispersibility, and the addition of NFC could effectively improve the stability of CNTs in the SPI solution.

### 3.5. Opacity Analysis

**Figure 5:**
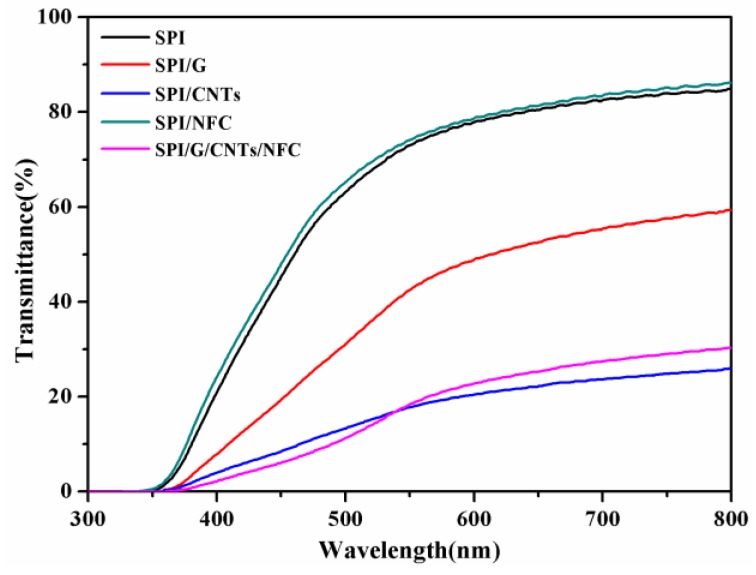
Ultraviolet–visible (UV–vis) spectroscopy of SPI film and SPI-based nanocomposites.

UV-vis spectroscopy was applied to study the effect of graphene, CNTs and NFC on the optical transmittance of different SPI-based films. As shown in Figure 5, the SPI/G film showed a lower transmittance than the control film, which was due to the absorption and light scattering of the graphene nanoplatelets. This result was consistent with the fact that graphene can partly absorb visible light [38]. The SPI/G/CNT/NFC nanocomposite film had a relatively low optical transmittance value, which could be explained that CNTs were dispersed uniformly and separately in the SPI matrix [39]. These results demonstrated that the SPI-based nanocomposite films containing the nanofillers of graphene, CNTs, and NFC, possessed superior barrier abilities for ultraviolet and visible light.

### 3.6. Mechanical Properties

**Figure 6:**
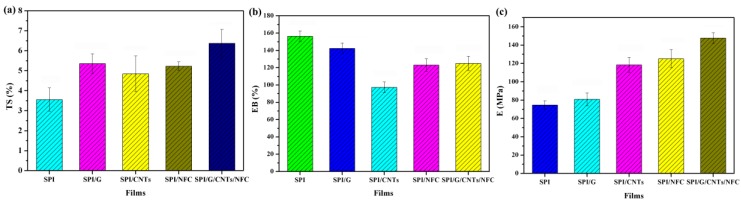
The mechanical properties of (**a**) tensile strength (TS), (**b**) elongation at break (EB), and (**c**) Young’s modulus (E) of SPI-based nanocomposite films.

The mechanical performances of the SPI-based nanocomposite films were evaluated by measuring the tensile strength (TS), elongation at break (EB), and Young’s modulus (E), as shown in Figure 6. The detailed data is listed in Table 2. Due to the relatively weak phase interfacial adhesion, the neat SPI film generally exhibited poor mechanical properties. In comparison with the neat film, the TS and E of the SPI/G film prepared from the aqueous graphene dispersion increased from 3.56 MPa to 5.36 MPa and from 74.7 MP to 80.8 MP, respectively. The enhancement was mainly attributed to the hydrogen bonding and π–π interaction between graphene nanosheets and SPI molecular chains [2]. With the addition of CNTs, the TS of the SPI/CNT film increased to 4.85 MPa, counting an increase of 36.2% compared to the unmodified SPI film. However, the EB of the SPI/CNT film decreased to 97.36 MPa, suggesting a reduction in the elasticity of the SPI-based nanocomposite film. This result suggested that the alignment of CNTs in the SPI matrix could have an influence on the interfacial stress transfer of hybrid film, resulting in an increase in chain brittleness and stiffness [39]. For the SPI/NFC film, the TS and E values increased to 5.23 MPa and 125.2 MPa, respectively. This improvement was possibly due to the formation of an NFC network within the SPI matrix [26]. The entanglement of cross-linked chains could maximize the interfaces available for stress transfer and limit the large deformation of the composite structure, thus increasing the toughness and flexibility of the films [30].

In particular, it is noteworthy that the SPI/G/CNT/NFC film yielded the highest TS (6.37 MPa) and E (147.7 MPa) values. Compared with the nanocomposites with improved mechanical performance via positive–negative charge interaction in our previously published paper [21], the EB and E values of the obtained nanocomposites in this study were approximately 1.5 times higher. This remarkable reinforcement of mechanical properties for the SPI/G/CNT/NFC nanocomposite film was mostly attributed to the synergistic effect from graphene, CNTs, and NFC within the SPI matrix [18]. The graphene nanosheets stably dispersed in the SPI matrix could absorb pristine CNTs through π–π stacking interaction, resulting in a more stable dispersion of CNTs in the SPI matrix [40]. Meanwhile, the NFC contributed to a highly dispersed state for the graphene and CNTs in the polymer matrix [27]. This uniform dispersal could strengthen the cross-linked framework and transfer the impulsive force across the nanocomposite film [16].

We also compared the mechanical behaviors of SPI/G/CNT/NFC film with other filler-reinforced SPI composite films. As summarized in Table 3, some strategies for fabricating SPI-based composite films often result in relatively low toughness. For instance, the SPI/MMT nanocomposites possessed TS as high as 7.32 MPa, but the corresponding EB decreased to 23.4% due to the lack of synergistic interface interactions [26]. The SPI/G/PEI-CNC composite film showed a TS value of 7.49 MPa, however, the EB was only 87.14% [21]. The low strain could also be observed in SPI/CMKGM [41], SPI/genipin [42], and SPI/SNC films [25]. In particular, the strength and strain of SPI/HNT film were comparable to those of SPI/G/CNT/NFC film, but its EB value was slightly higher because of the entanglement and penetration between HNTs and SPI segments [7]. Notably, the SPI-based composites reinforced with relatively low content of G/CNT/NFC exhibited a favorable combination of strength and flexibility.

**Figure 7:**
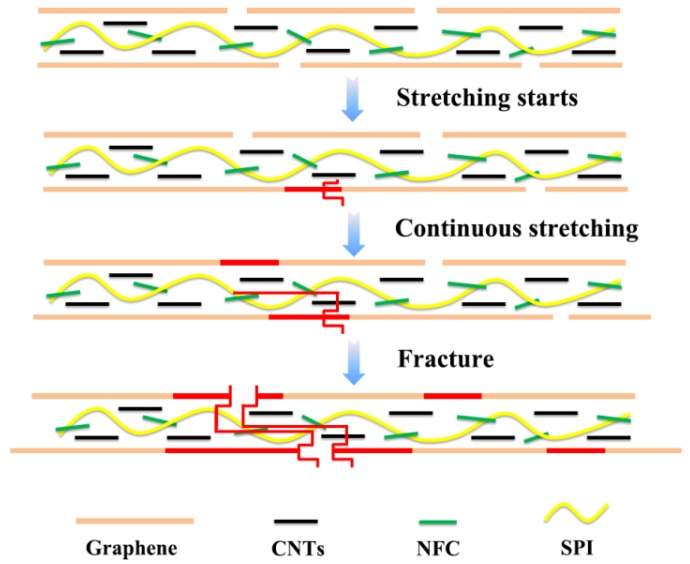
Proposed crack extension model of SPI/G/CNT/NFC nanocomposites.

To understand the synergistic effect from graphene, CNTs, and NFC, a crack extension model was proposed, as shown in Figure 7. When the loading was gradually applied to SPI/G/CNT/NFC nanocomposite film, the hydrogen bonds were firstly destroyed, and relative slippage started to occur between the adjacent graphene nanosheets, initiating the crack propagation. The friction between graphene nanosheets and CNTs then led to the movement of the CNT along the graphene nanosheets. In addition, 1D nanofibrous NFC could act as extensional tentacles for the hybrid structures and was entangled with the polymer chains, which increased more friction between graphene and CNT nanosheets. The SPI chains interpenetrated into the interlayer and provided enough interactions between adjacent nanofillers. With further increasing deformation, the covalent bonds between NFC and SPI molecular chains were damaged, resulting in much more energy absorption. This cycling of initiation–propagation–deflection led to the ultimate fracture of the SPI/G/CNT/NFC nanocomposite films. The process also resulted in higher dissipation of energy and therefore the SPI/G/CNT/NFC nanocomposite films achieved higher strength.

### 3.7. Thermal Performance

**Figure 8:**
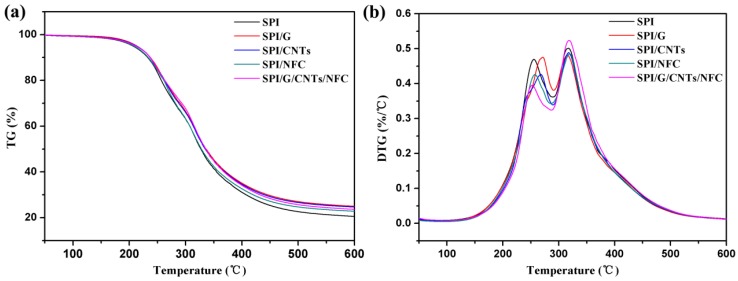
(**a**) Thermogravimetric analysis (TGA) and (**b**) differential thermogravimetry (DTG) curves of SPI, SPI/G, SPI/CNT, SPI/NFC, and SPI/G/CNT/NFC series films.

The thermal properties of SPI-based nanocomposite films were examined by thermogravimetric analysis (TGA) (Figure 8). The SPI hybrid films represented two degradation stages, as shown in Figure 8b. The first stage (130–280 °C) was related to the weight loss of glycerol [44]. The second stage (280–420 °C) was mainly associated with the SPI backbone decomposition [33]. The detailed data is listed in Table 4. It was noteworthy that the residual mass (25.0 wt %) of SPI/G/CNT/NFC film at 550 °C was higher than that of pristine SPI film (21.9 wt %), suggesting the increased thermal stability of the nanocomposite film. This enhancement might be due to the high aspect ratio and thermal conductivity of carbon-based fillers which could promote thermal transport across the polymer-filler interfaces and increase the temperature of backbone peptide degradation [43]. In addition, the favorable thermal performance of the SPI-based nanocomposite film was also attributed to the cross-linking hierarchical structure and multiple interactions between the fillers and SPI matrix, as confirmed by the ATR–FTIR and XRD analysis.

### 3.8. Surface Hydrophilicity Analysis

**Figure 9:**
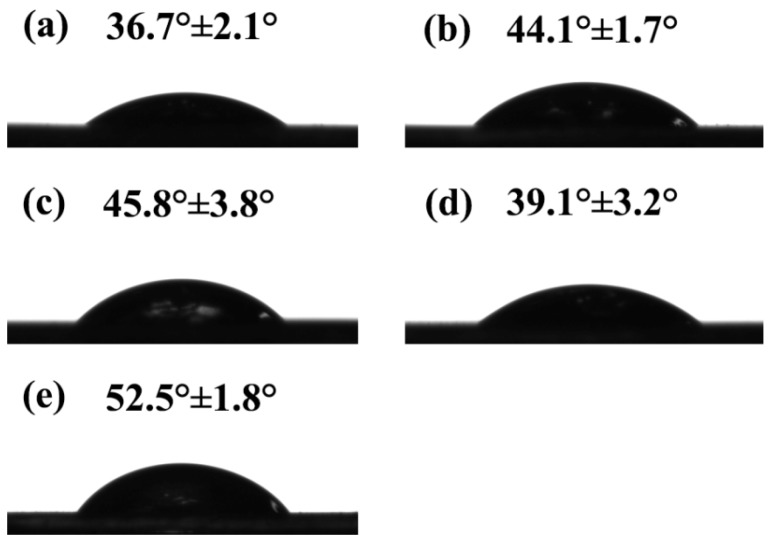
WCA values of (**a**) SPI, (**b**) SPI/G, (**c**) SPI/CNT, (**d**) SPI/NFC, and (**e**) SPI/G/CNT/NFC series films.

Surface-water contact angle measurements were carried out to investigate the surface hydrophilicity of the SPI-based nanocomposite films. As shown in Figure 9, the neat SPI film displayed a relatively low WCA value of 36.7° because of its hydrophilicity nature [9]. The WCA values of the SPI/G and SPI/CNT films increased to 44.1° and 45.8°, respectively, demonstrating an increase in surface hydrophobicity as compared to the unmodified SPI film. The SPI/NFC film showed a lower WCA value of 39.1° than the SPI/G and SPI/CNT films, which might be due to the higher content of hydrophobic groups on the NFC-modified film surface [45]. In particular, the SPI/G/CNT/NFC composite film showed the highest WCA value of 52.5° with an average increase of 43%, as compared to the neat SPI film. This feature could be attributed to the increased hydrogen bonds and polar groups in the cross-linked polymer network, which led to the improvement in the surface hydrophobicity of the SPI nanocomposite film [21].

### 3.9. Water Resistance

**Figure 10:**
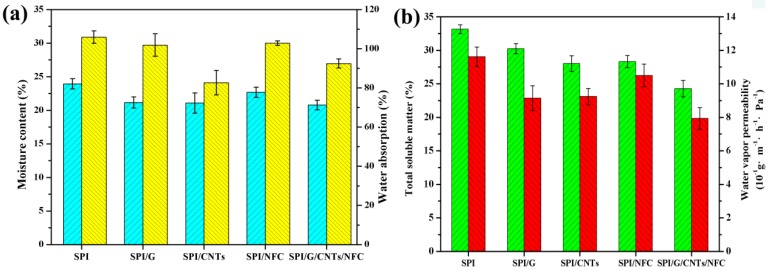
(**a**) Moisture content (MC) and water absorption (WA), (**b**) total soluble matter (TSM) and water vapor permeability (WVP) of pristine SPI film and modified SPI nanocomposite films.

The water resistance of the SPI-based nanocomposite film was determined by testing the moisture content (MC), total soluble matter (TSM), and water absorption (WA). As shown in Figure 10, the neat SPI film generally possessed poor water-resistance properties. After the modification of the graphene and CNTs, the MC and WA values of the films simultaneously decreased, which demonstrated that the SPI/G and SPI/CNT films had higher resistance to water absorption than the unmodified film. Compared with the SPI film, the TSM and WA of the SPI/G/CNT/NFC film obviously decreased to 24.28% (26.78% decrease) and 92.42% (12.73% decease), respectively. These results suggested that there could exist synergetic effects among the graphene, CNTs, and NFC. The combination of these could promote the formation of hydrogen bonds and the cross-linking network in the composite, and effectively prevent the swelling of the polymeric matrix in wet conditions [46].

The water vapor permeability (WVP) was measured to determine the water vapor barrier abilities of the nanocomposite films. Compared with neat SPI film, the WVP values of SPI/G, SPI/CNT and SPI/NFC film were decreased by 21.26%, 20.48% and 9.64%, respectively, which was possibly due to the tortuosity effect caused by the high aspect ratio of graphene, CNTs and NFC [42]. Noticeably, the SPI/G/CNT/NFC film exhibited the lowest WVP value of 7.93% (31.76% decrease) among all the films, suggesting its excellent water vapor barrier property. This remarkable improvement indicated the synergetic effect from graphene, CNTs and NFC. The combination of these might limit the molecules mobility of the SPI chains and lead to a reduction in the permeation of water vapor under a denser cross-linked network [41]. Therefore, this SPI/G/CNT/NFC nanocomposite film showed superior water resistance and great potential in practical applications.

## 4. Conclusions

In the present study, we fabricated nacre-like SPI-based nanocomposites with the assembly of 2D graphene and 1D CNTs and NFC via a layer-by-layer self-assembly process. The combination of covalent bonds, hydrogen bonding, and π–π stacking in the mixture exhibited a synergistic strengthening effect on the mechanical properties and water resistence of the SPI-based nanocomposites. The TS of the SPI/G/CNT/NFC film increased from 3.56 MPa to 6.37 MPa, and the TSM and WVP decreased by 26.78% and 31.76%, respectively, compared with the unmodified one. In addition, the cross-linking network of SPI/G/CNT/NFC and multiple interface interactions between the nanophase and SPI matrix endowed the composite films with better thermal stability than the control. The biomimetic strategy proposed in this work represented a promising approach for preparing high-performance composites.

## Figures and Tables

**Table 1:** Experimental details and summary of the soy protein isolate (SPI) based films.

Codes	SPI (g)	Glycerol (g)	Water (g)	Graphene (G) (g)	CNTs (g)	Nanofibrillated Cellulose (NFC) (g)
SPI	5	2.5	95	-	-	-
SPI/G	5	2.5	-	95	-	-
SPI/carbon nanotubes (CNT)	5	2.5	95	-	0.01	-
SPI/NFC	5	2.5	95	-	-	1
SPI/G/CNT/NFC	5	2.5	95	95	0.01	1

**Table 2:** The thickness, TS, EB, and E values of SPI-based nanocomposite films.

Type	Thickness	TS	EB	E
	(mm)	(MPa)	(%)	(MPa)
SPI	0.226 (0.014)	3.56 (0.58)	156.36 (6.16)	74.7 (4.62)
SPI/G	0.228 (0.009)	5.36 (0.49)	142.26 (6.25)	80.8 (7.08)
SPI/CNT	0.249 (0.021)	4.85 (0.89)	97.36 (6.19)	118.4 (8.17)
SPI/NFC	0.251 (0.024)	5.23 (0.21)	123.13 (7.25)	125.2 (9.87)
SPI/G/CNT/NFC	0.222 (0.013)	6.37 (0.70)	124.91 (8.24)	147.7 (5.81)

The values in parenthesis represent the standard deviation.

**Table 3:** Comparison of TS and EB of SPI/G/CNT/NFC nanocomposite film with other SPI-based composite films in previous literature.

Ref	Fillers ^a^	Filler Content Based on SPI (wt %)	TS (MPa)	EB (%)
[43]	EGDE/CNC	2	4.79	86.5
[42]	Gen	5	4.60	12.14
[26]	MMT	7.5	7.32	23.4
[21]	G/PEI-CNC	0.4	7.49	87.14
[41]	CMKGM	25	12.50	5.21
[7]	HNTs	4	6.7	142
[25]	SNC	40	5.08	21.35
This work	G/CNTs/NFC	0.4	6.37	124.91

**^a^** In this column, EGDE/CNC = ethyleneglycol diglycidyl ether/cellulose nanocrystals; Gen = genipin; MMT = montmorillonite; G/PEI-CNC = graphene/polyethyleneimine; CMKGM = carboxymethyl konjac glucomannan, modified cellulose nanocrystals; HNTs = halloysite nanotubes; SNC = starch nanocrystals.

**Table 4:** The TGA data of SPI, SPI/G, SPI/CNT, SPI/NFC, and SPI/G/CNT/NFC nanocomposite films.

Films	*T*_max1_ (°C)	*T*_max2_ (°C)	Residual Mass (wt %) at 550 °C
SPI	252.77	314.67	21.9
SPI/G	271.56	315.30	25.8
SPI/CNT	264.06	313.79	25.5
SPI/NFC	253.74	316.21	24.3
SPI/G/CNT/NFC	249.57	317.17	25.0

*T*_max_: temperature at maximum degradation rate.
